# An integrated metabolome and transcriptome approach reveals the fruit flavor and regulatory network during jujube fruit development

**DOI:** 10.3389/fpls.2022.952698

**Published:** 2022-09-23

**Authors:** Dongye Lu, Lei Zhang, Yang Wu, Qinghua Pan, Yuping Zhang, Ping Liu

**Affiliations:** ^1^Beijing Academy of Agriculture and Forestry Sciences, Institute of Forestry and Pomology, Beijing, China; ^2^State Key Laboratory of Tree Genetics and Breeding, Key Laboratory of Tree Breeding and Cultivation of National Forestry and Grassland Administration, Research Institute of Forestry, Chinese Academy of Forestry, Beijing, China; ^3^Research Center of Chinese Jujube, Hebei Agricultural University, Baoding, China

**Keywords:** *Ziziphus jujuba*, metabolome, transcriptome, fruit quality, regulatory network

## Abstract

The fruit flavor is a key economic value attribute of jujube. Here we compared metabolomes and transcriptomes of “Mazao” (ST) and “Ping’anhuluzao” (HK) with unique flavors during fruit development. We identified 437 differential metabolites, mainly sugars, acids, and lipids. Fructose, glucose, mannose and citric acid, and malic acid are the determinants of sugar and acid taste of jujube fruit. Based on the transcriptome, 16,245 differentially expressed genes (DEGs) were identified, which were involved in “glucosyltransferase activity,” “lipid binding,” and “anion transmembrane transporter activity” processes. Both transcriptome and metabolome showed that developmental stages 2 and 3 were important transition periods for jujube maturation. Based on WGCNA and gene-metabolite correlation analysis, modules, and transcription factors (*ZjHAP3*, *ZjTCP14*, and *ZjMYB78*) highly related to sugar and acid were identified. Our results provide new insights into the mechanism of sugar and acid accumulation in jujube fruit and provide clues for the development of jujube with a unique flavor.

## Introduction

*Ziziphus jujuba* belongs to *Ziziphus* of Rhamnaceae, which is native to China. It is rich in germplasm resources and has a long history of cultivation. There are many cultivars of jujube, which can be traditionally divided into three categories according to their uses: dried, fresh, dried, and fresh jujube. Among them, the production of dried fruits is the largest in China, while fresh jujube is abundant in nutrition and tastes crisply (Liu and Wang, 2009). Studies have shown that jujube is rich in carbohydrates, cyclic adenosine monophosphate, triterpenoids, flavonoids, vitamin compounds, and inorganic salts such as phosphorus, calcium, and iron, which have high nutritional and medicinal value (Li et al., 2007; Gao et al., 2013). Understanding the differences and dynamic changes in nutritional components of jujube fruits during ripening will provide valuable information for the genetic improvement of jujube.

Flavor quality is an important economic attribute of fruits that affects people’s choices (Barrett et al., 2010; Goldenberg et al., 2018). The acid, sugar composition and content of fruits determine important factors of fruit flavor (Zhu et al., 2018). In the process of jujube domestication, the sweetness/acidity of jujube fruit is based on the genetic selection that determines the content of acid and sugar (Huang et al., 2016). The dynamic analysis of sugar components in jujube fruits showed that fructose and glucose were the main accumulations in the early stages of fruit accumulation, while sucrose was dominant in the later stages (Zhang et al., 2021). Zhao et al. (2021) revealed the content characteristics of organic acid components in the fruits of 219 jujube germplasm and found that the contents of malic, quinic, and citric acids in jujube fruits were in the top three. Glucose metabolism produces pyruvate through glycolysis, which enters the tricarboxylic acid (TCA) cycle to form citric acid, malic acid, and others. The sugar content of jujube fruits is significantly higher than that of wild jujube and other fruit trees, such as apples, peaches, and grapes (Huang et al., 2021). Compared with Rosales fruit, the gene families involved in glucose metabolism in the jujube genome have a higher degree of expansion (Liu et al., 2014). The sugar content in fruits largely depends on the balance between the sugar source and the sink (Huang et al., 2021). Therefore, it is of great significance to reveal the metabolite contents of sugars and organic acids in jujube during different development processes as well as the biosynthetic pathways and regulatory mechanisms affecting their accumulation.

With the continuous development of omics technology, metabolome and transcriptome analysis have been successfully applied to study the regulatory mechanisms of leaf color, fruit anthocyanin, flavonoids, and other nutrients accumulation in jujube and apples (Shi et al., 2020; Xu et al., 2020; Li et al., 2021). In addition, the betaine biosynthetic pathway determines the pitaya fruit color formation including peel color (red and yellow) and the pulp color (Zhou et al., 2020). Gong et al. (2021b) revealed the differences in sugar accumulation between cultivated and wild watermelon through transcriptomics and metabolomics and found that UDP-glycosyltransferase was closely associated with glycosylation of cucurbitacin. By combining the results of WGCNA and metabolomics, Chu et al. (2022) identified genes and metabolites for flesh sweetness, bitterness, and color of watermelon. Xiong et al. (2020) analyzed the accumulation patterns of sugars, organic acids, ascorbic acid, and related genes throughout the development of yellow kiwifruit. Yang revealed the expression patterns of sugar, acid, flavonoid metabolites and genes during cherry ripening (Yang et al., 2021). Although we have studied fruit quality at the level of transcription and metabolism, jujube flavor, as a complex trait, still varies significantly among cultivars, so we need to explore its molecular mechanism.

In this study, we sequenced the metabolome and transcriptome datasets of “Mazao” (ST) and “Ping’anhuluzao” (HK) jujube cultivars at 30, 60, 80, 100, and 110 days after anthesis. These two cultivars have good flavor and rich nutrients. The dynamic accumulation patterns of sugars, organic acids, fatty acids and other nutrients at five developmental stages were analyzed for their primary metabolome, and gene expression patterns were analyzed by transcriptomics, to explore possible regulatory genes affecting jujube flavor by joint analysis. This study provided a rich genetic basis for further enriching the flavor of jujube fruits.

## Materials and methods

### Plant materials

**Figure 1:**
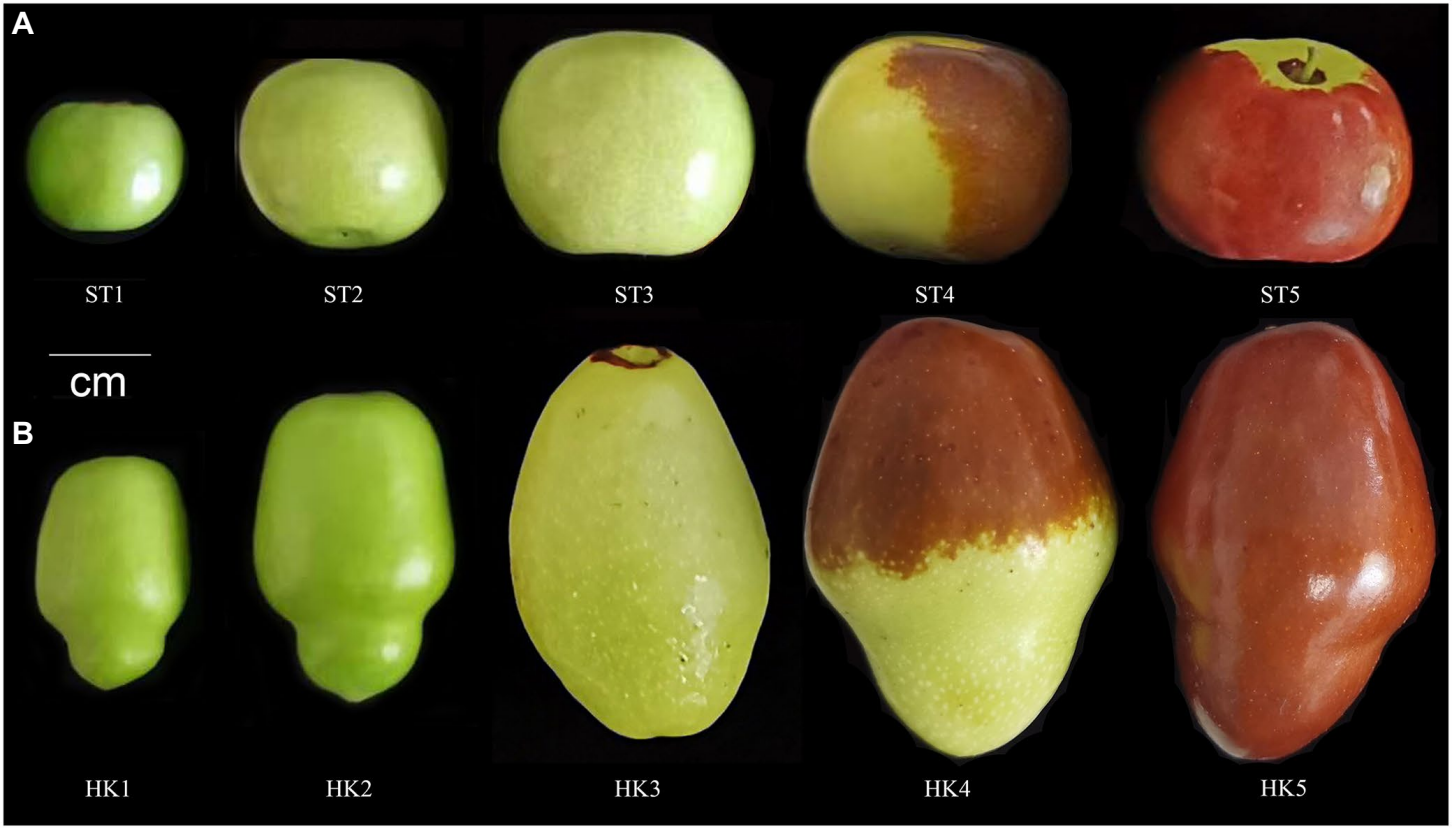
Phenotype of five developmental stages in “Mazao” **(A)** and “Ping’anhuluzao” **(B)**.

“Mazao” (ST) and “Ping’anhuluzao” (HK) were excellent new cultivars selected in recent years for live breeding (Lu et al., 2022). Among them, ST jujube is flat and round while HK possesses constricted type (Figure 1), HK accumulates high total soluble sugar contents (22.68%) and low total organic acid contents (0.76 g/kg) at maturity, while ST was the opposite of its, with total soluble sugar contents of 14.71% and total organic acid contents of 1.04 g/kg. The trees were cultivated under normal field conditions, including irrigation, fertilization, and disease and pest control. The fruits of HK and ST were collected from the town of Qinglonghu (116°5′E, 39°47′N), Fangshan District, Beijing, China in 2021 at five different periods of 30 (young), 60 (enlarged), 80 (white-ripened), 100 (half-red), and 110 (full-red) days after anthesis. Fruits were pitted and chopped, then rapidly placed in liquid nitrogen and stored at −80°C until used for metabolomic analysis and transcriptomic sequencing. Three biological replicates were taken from each period of the two cultivars.

### UPLC–MS/MS system-based widely targeted metabolomics analysis

The primary metabolites were extracted and identified by Metware Biotechnology Co., Ltd.^1^ Biological samples were freeze-dried using a vacuum freeze-dryer (Scientz-100F), and 100 mg of the powder was dissolved in 1.2 ml of 70% methanol solution and kept at 4°C overnight. The filtered extracts were used for metabolite profiling by UPLC–MS/MS system (Applied Biosystems 4500 Q TRAP) analysis and quantification was performed by multiple reaction monitoring (MRM) in a triple quadrupole spectrometer (Chen et al., 2013). Metabolites were identified by comparing the exact mass, fragmentation patterns, and retention times with the standards from a self-compiled database (MetWare, Wuhan, China) (Chen et al., 2013).

### RNA extracted and RNA-sequencing

Total RNA was extracted from fruits (HK1, HK2, HK3, HK4, HK5, ST1, ST2, ST3, ST4, and ST5 with three biological replicates) using the RNAprep Pure Plant Plus Kit (TIANGEN, Beijing, China). A total amount of 1 μg RNA per sample was used for the sequenced library by NEBNext^®^ UltraTM RNA Library Prep Kit for Illumina^®^ (NEB, United States). The cDNA library products were sequenced by the Illumina Hiseq platform with 125 bp/150 bp paired-end reads. The raw data was filtered using fastp v 0.19.3 (Chen et al., 2018), mainly removing reads with adapters; when any sequencing read contained more than 10% of the bases of the read, the paired reads were removed; when any sequencing read contained more than 50% of the bases of the read with low quality (*Q* ≤ 20), the paired reads were removed. Clean reads were compared to a reference genome (*Ziziphus jujuba* Mill. “Dongzao”) using HISAT v2.1.0 (Liu et al., 2014). Novel gene prediction was performed using StringTie v1.3.4d (Pertea et al., 2015). Feature Counts v1.6.2 (Liao et al., 2014) was used to calculate the gene alignments and FPKM.

### Differential metabolites and genes analysis

Unsupervised principal component analysis (PCA) was performed by statistics function “prcomp” within R v4.1.2. variable importance in projection (VIP) values were extracted from OPLS-DA result by using the R package “MetaboAnalystR” (Chong and Xia, 2018). Metabolites with VIP ≥ 1 and log_2_(fold change) ≥1 were considered significantly differential accumulation metabolites (DAMs) between groups (HK2 vs. HK1, HK3 vs. HK2, HK4 vs. HK3, HK5 vs. HK4, ST2 vs. ST1, ST3 vs. ST2, ST4 vs. ST3, and ST5 vs. ST4). To analyze the changing trend of metabolites, DAMs were standardized (*z*-score) and clustered by K-means.

DESeq2 v1.22.1 (Love et al., 2014) was used to analyze the differential expression genes (DEGs) with |log_2_FC(fold change)| ≥ 1 and *p*-value <0.05 (Varet et al., 2016). The functions of the unigenes were annotated by the NR, KOG, SwissProt, GO, and KEGG databases (Ashburner et al., 2000; Bairoch and Apweiler, 2000; Kanehisa and Goto, 2000; Natale et al., 2000; Ogata et al., 2000; Wilke et al., 2012).

### Combined metabolome and transcriptome analysis

The quantitative values of genes and metabolites in all samples were used for correlation analysis. The “cor” function in R was used to calculate the Pearson correlation coefficient of genes and metabolites with an absolute threshold larger than 0.85 and a *p*-value <0.05. The correlation analysis results of different genes and metabolites were selected. Differential genes and differential metabolites in each pathway were analyzed by CCA (canonical correlation analysis) (González et al., 2008). WGCNA v1.69 was used for weighted gene co-expression network analysis (WGCNA). Before WGCNA analysis, the genes with FPKM <0.1 were filtered out from all samples. Pearson’s correlation, calculation of soft-power threshold (β), and the division of modules were performed according to previous studies (Chen et al., 2021; Lu et al., 2022), in this study, soft-power threshold (β) was set to 7, the minimum number of genes contained in the modules was set to 50, while the threshold for merging similar modules was set to 0.25. Cytoscape 3.8 was used for visualization of the control network with default settings (Otasek et al., 2019).

### qRT-PCR

Ten DEGs were selected for qRT-PCR analysis, and *ZjUBQ* was used as the internal reference gene. The primers were listed in Supplementary Table S2. The RNA was extracted from jujube fruit as described above. qRT-PCR was performed using TB Green^®^ Premix Ex Taq™ II (Takara, Beijing, China). Three technical replicates and three biological replicates were performed. The relative expression levels were calculated using the 2^−ΔΔCt^method (Livak and Schmittgen, 2001).

## Results

### Overview of metabolite accumulation patterns during jujube fruit development

To define a comprehensive landscape of metabolite profile during fruit development of HK and ST, we performed metabolite profiling by using LC-MS. During fruit ripening, the pericarp changed from green to yellow and red, and flavonoids accumulated rapidly. A total of 508 metabolites of 10 categories were obtained at different development stages of jujube fruits, including organic acids, amino acids and derivatives, saccharides and alcohols, free fatty acids, nucleotides and derivatives, lysophosphatidyl cholines (LPCs), lysophosphatidyl ethanolamines (LPEs), vitamins, glycerol esters, and sphingolipids (Supplementary Table S1).

**Figure 2:**
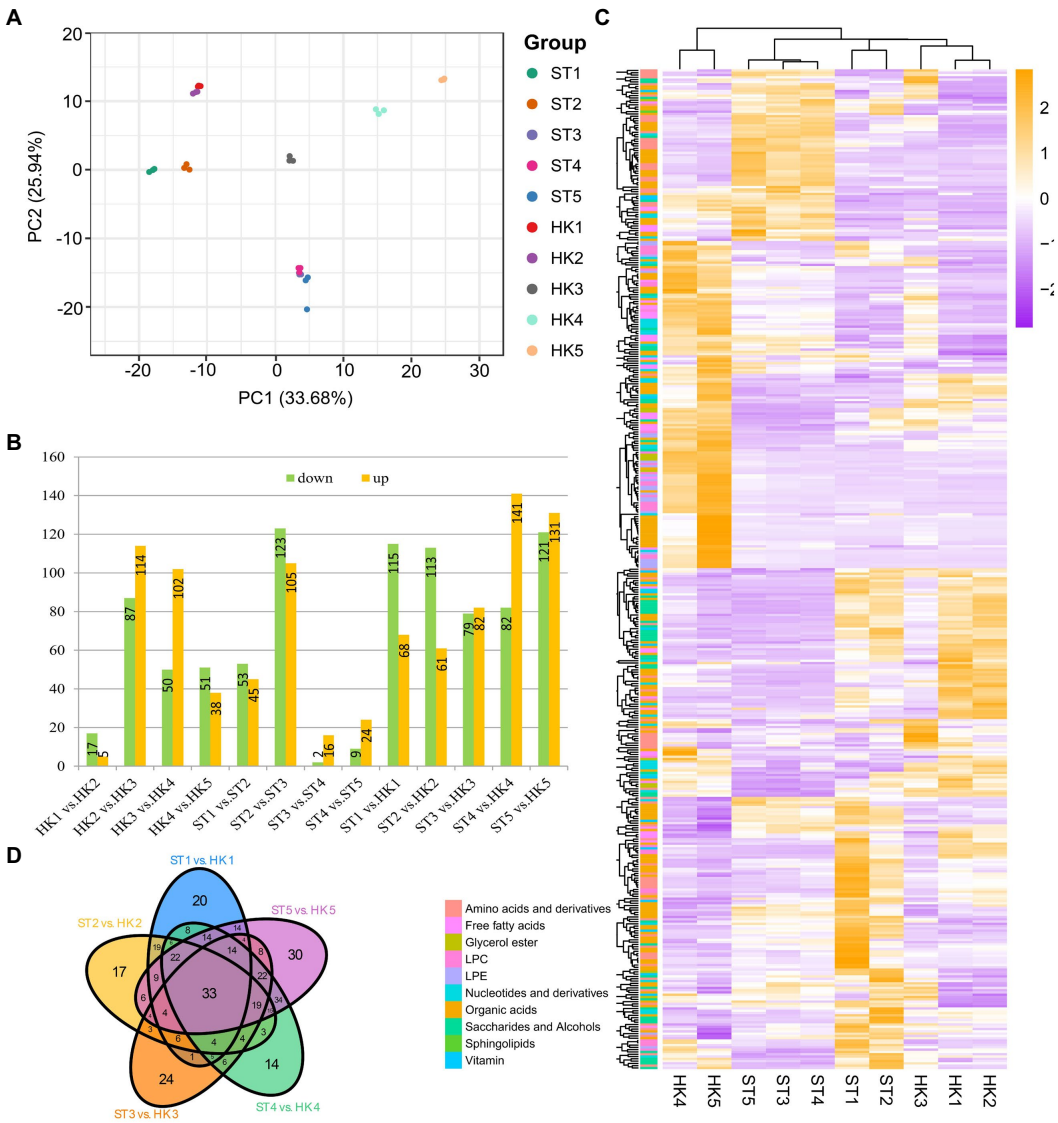
Comparison of metabolites in different developmental stages of jujube fruit. **(A)** Principal component analysis (PCA) score plot of all metabolites in 30 samples. HK1 (ST1), HK2 (ST2), HK3 (ST3), HK4 (ST4), and HK5 (ST5) represent the samples at 30, 60, 80, 100, and 110 days after anthesis, respectively. **(B)** The number of differentially accumulation metabolites (DAMs) by comparing HK1 vs. HK2, HK2 vs. HK3, HK3 vs. HK4, HK4 vs. HK5, ST1 vs. ST2, ST2 vs. ST3, ST3 vs. ST4, ST4 vs. ST5, ST1 vs. HK1, ST2 vs. HK2, ST3 vs. HK3, ST4 vs. HK4, ST5 vs. HK5. **(C)** Overview of DAMs of two cultivars in five periods. **(D)** Venn diagram of the number of different developmental stages and cultivars.

Principal component analysis was used to analyze the data for all compounds from five developmental stages for two cultivars with three biological replicates; the objective was to provide a preliminary understanding of the overall metabolic differences between groups of samples including different fruit development stages and cultivars and the magnitude of variability between samples within groups. PC1 and PC2 explained 33.68% and 25.94% of the variation, respectively (Figure 2A). The results showed that the variation between different fruit development stages was greater than the variation between the two cultivars. In addition, there was a large gap between the metabolomes of the third stage and the other four stages of development.

### Analysis of metabolite differences and K-means analysis during fruit ripening in jujube

Heatmap and cluster analysis yielded an overview of dynamic metabolome changes during fruit development. To further explore the metabolic differences in the developmental stages and between cultivars, we conducted a different analysis. A total of 437 differential accumulation metabolites (DAMs) were identified (Figures 2B,C), with 308 DAMs in HK, 289 DAMs in ST, and 392 DAMs between HK and ST. There were more differential metabolites in HK3 vs. HK2 and ST3 vs. ST2, which was consistent with the results of PCA. In other words, the shift from stage 2 to 3 was an important transition in jujube fruit development. There were 33 common differential metabolites between the two cultivars at different periods, including 18 organic acids, eight amino acids and derivatives, two nucleotides and derivatives, two LPCs, one glycerol ester, one saccharide and alcohol, and one vitamin (Figure 2D).

**Figure 3:**
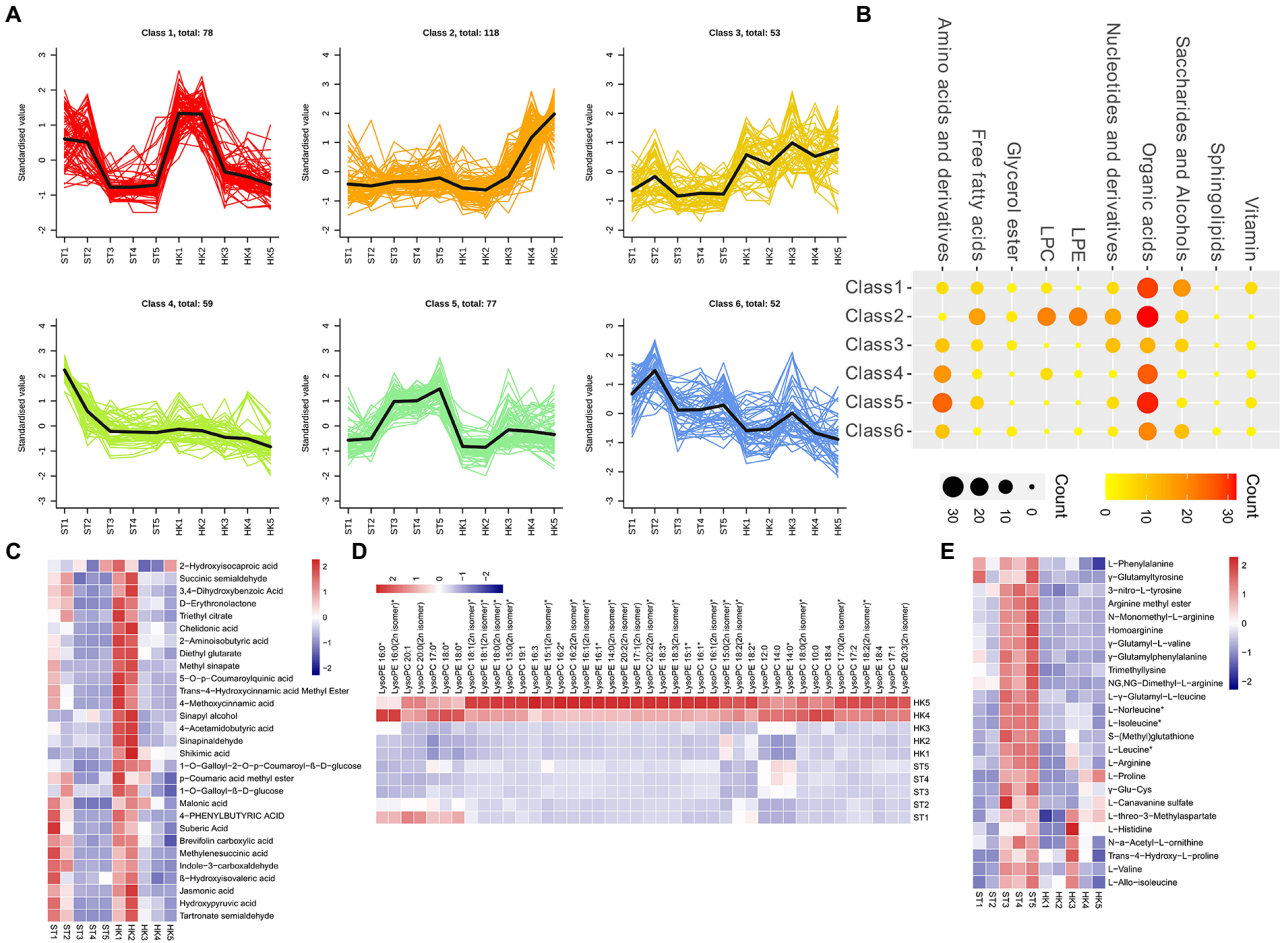
Cluster analysis of differential metabolites. **(A)** K-means cluster analysis of differential metabolites. **(B)** The number of metabolites in each category. Nodes from small to large and light to dark represent the number of metabolites. The accumulation pattern metabolites of class 1 **(C)**, 2 **(D)**, and 5 **(E)**.

To analyze the trends in metabolite content throughout fruit development, the relative contents of all the different metabolites identified in all group comparisons were standardized according to the screening criteria and then subjected to K-means cluster analysis (Figure 3A). Class1 contained 78 DAMs (organic acids, saccharides, alcohols, etc.) that accumulated mainly during the early stage (1, 2), and were reduced during the later stages (3, 4, 5) of fruit development (Figures 3B,C). The DAMs of Class 2 were mainly concentrated in stages 4 and 5 of HK. It contained a large number of organic acids and almost all lipids (LPC and LPE), which were specifically high in HK (Figures 3B,D) 0.77 metabolites, such as amino acids and derivatives and organic acids in Class 5, accumulated in large amounts in ST fruit at later stages (3, 4, 5), which is opposite to the metabolite accumulation mode in Class 2 (Figures 3B,E). This showed that the accumulation patterns of organic acids and saccharides were similar in both cultivars, but there were significant differences in lipids and amino acids, which may lead to the different nutritional value and taste of jujube.

### Accumulation pattern and correlation analysis of sugars and organic acids in jujube fruits

**Figure 4:**
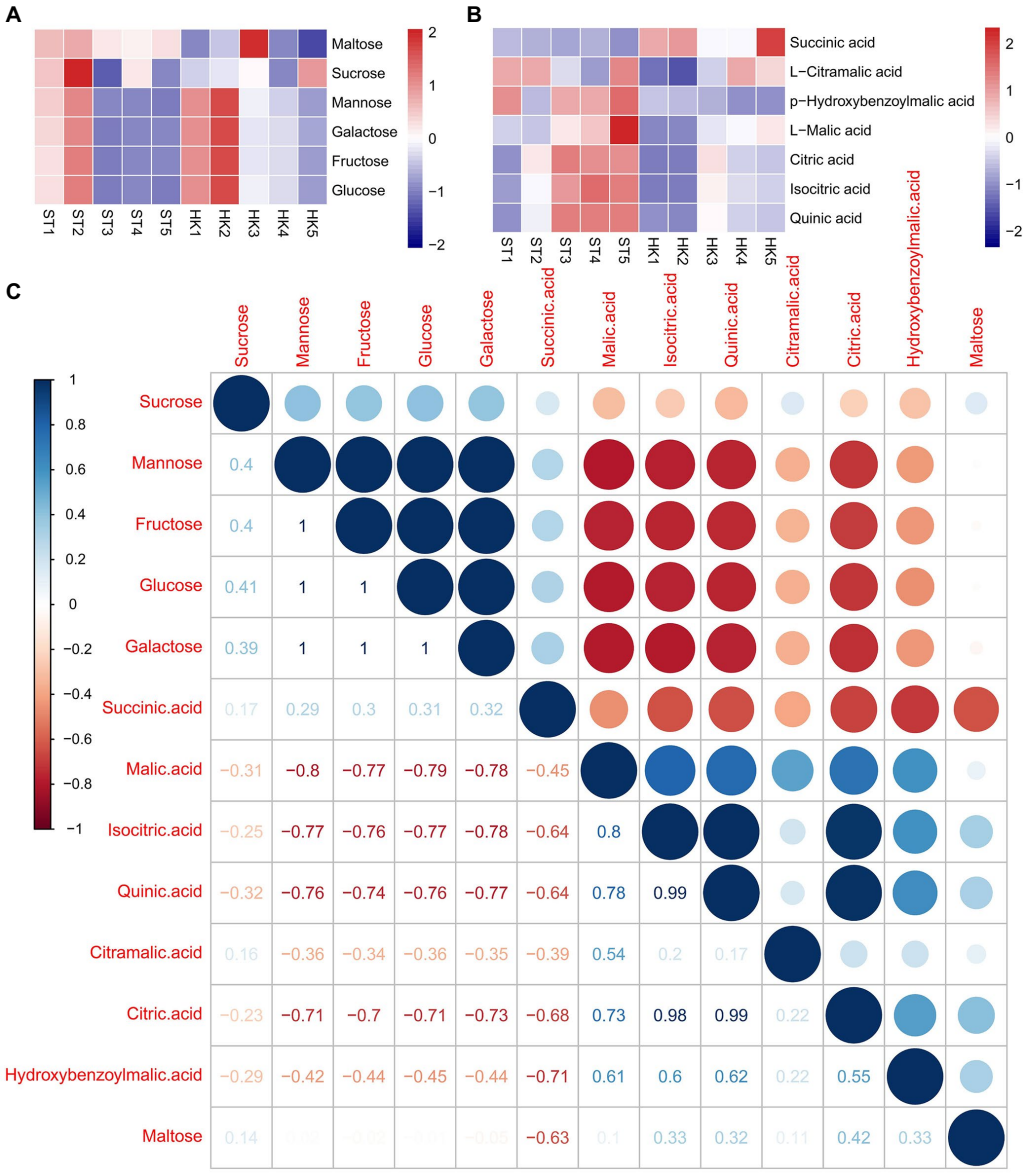
Patterns of sugar and acid accumulation and their correlation in jujube. Accumulation patterns of six major sugars **(A)** and seven major acids **(B)**. **(C)** Correlation between sugars and acids.

Sugar and acids are important factors affecting fruit flavor. We found 50 saccharides and alcohol metabolites with differential accumulation in either developmental stage or cultivar, among which fructose, glucose, mannose, and galactose were the main soluble sugars of jujube. The contents of four sugars showed similar trends in ST and HK, with higher contents in stages 1 and 2 and a decreasing trend in the later stages (Figure 4A). The results indicated that sugar accumulation, which determines fruit sweetness, mainly occurred in the early stages of fruit development. Furthermore, 151 kinds of organic acids were found, including the common soluble acids citric acid, malic acid and quinic acid. Citric acid and malic acids were found to be the most abundant. Their accumulation pattern was opposite to the trend of sugar content, and the content increased with fruit ripening. In addition, the organic acid content of ST was higher than that of HK (Figure 4B). To further explain the relationship between sugar and organic acids, the correlation between organic acids and sugar content was analyzed. The results showed that there was a positive correlation among the four sugars except for maltose. Citric acid, malic acid, and quinic acid were negatively correlated with sugars, while succinic acid was positively correlated with sugars (Figure 4C).

### Transcriptome analysis of the jujube fruit of two cultivars at different development stages

To further explore the possible regulatory genes affecting DAMs, we also sequenced the transcriptomes of jujube fruits at each stage. After removing the unknown reads, low-quality reads, and adaptor sequences, a total of 206.95 Gb clean data were obtained from 30 libraries with an average GC content of 43.88% (Supplementary Table S3). A total of 16,245 differentially expressed genes (DEGs) were identified from 13 differential comparisons (between different developmental stages and different cultivars). The number of DEGs between the comparison combinations ranged from 304 to 9,342, with ST3 vs. ST2 reaching a maximum of 9,342 DEGs (Supplementary Figure S1).

To reveal the molecular functions of DEGs, GO enrichment analysis indicated that they were more widely distributed in three categories of biological processes, molecular functions, and cell components. Multiple comparative combinations were enriched into the categories of “glucosyltransferase activity,” “lipid binding,” “anion transmembrane transporter activity,” “anion transport,” “photosynthesis,” “thylakoid” membrane” and “photosystem.” ST3 vs. ST2 contained the most DEGs, which were significantly enriched in “phosphatase activity,” “metal cluster binding,” “ribonucleoside binding.” DEGs of HK3 vs. HK2 were enriched in multiple cell structure related categories, including “supramolecular polymer,” “polymeric cytoskeletal fiber,” and “microtubule” (Supplementary Figure S2). KEGG enrichment indicated that DEGs were involved in starch and sucrose metabolism as well as secondary metabolites in periods 2 and 3. Among them, HK was uniquely enriched in fatty acid biosynthesis, metabolism, and degradation (Supplementary Figures S3, S4).

### Identification of WGCNA modules associated with fruit quality

**Figure 5:**
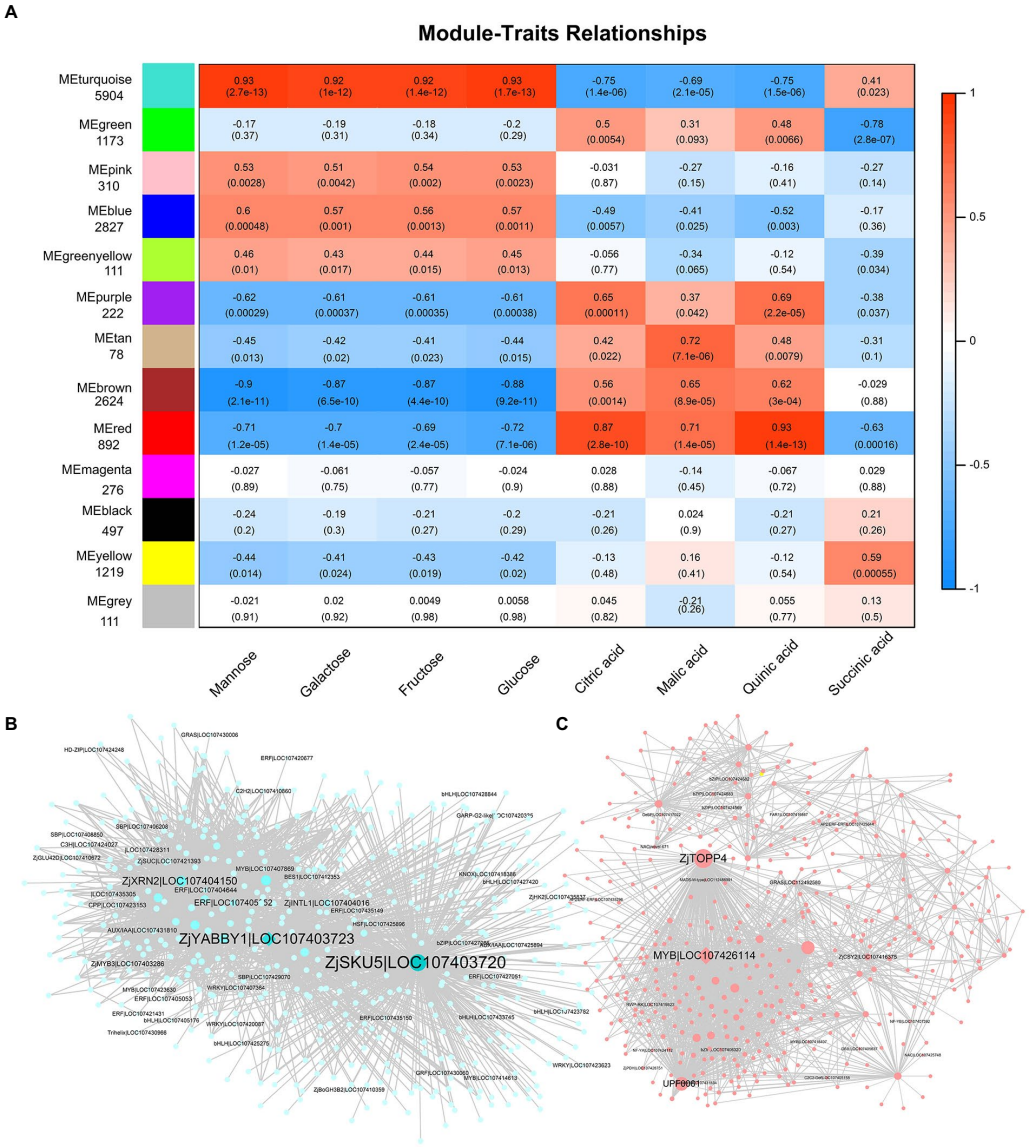
Weighted gene co-expression network analysis (WGCNA) of DEGs. **(A)** The relationship between modules and fruit quality. Weighted module-trait correlations and corresponding *p* values. The color scale on the right shows module-trait correlations from −1 (blue) to 1 (red). Cytoscape representation of co-expressed genes with edge weight ≥ 0.50 in the “turquoise” module **(B)** and ≥ 0.30 in the “red” module **(C)**.

To reveal potential relationships between genes and fruit quality, we performed the WGCNA on DEGs. The differential genes were divided into 13 modules (Figure 5A). The modules were related to sugars and acids in fruit. The turquoise module was positively correlated with the four main sugars and succinic acids (0.41–0.93) but negatively correlated with the levels of citric, malic, and quinic acid (−0.75 to −0.69). In addition, the brown and red modules were negatively correlated with sugar content and positively correlated with organic acid content. This further suggests that there is a negative correlation between sugars and acids (Figure 5A). Further analysis showed that *ZjSKU5* (monocopper oxidase-like protein SKU5, LOC107403720), *ZjYABBY1* (C2C2-YABBY, LOC107403723), *ZjTOPP4* (serine/threonine-protein phosphatase PP1-like, LOC107412332), and *ZjMYB78* (LOC107426114) are the core genes of the module turquoise and red (Figures 5B,C).

### Differentially expressed genes involved in sugar and organic acid metabolism

**Figure 6:**
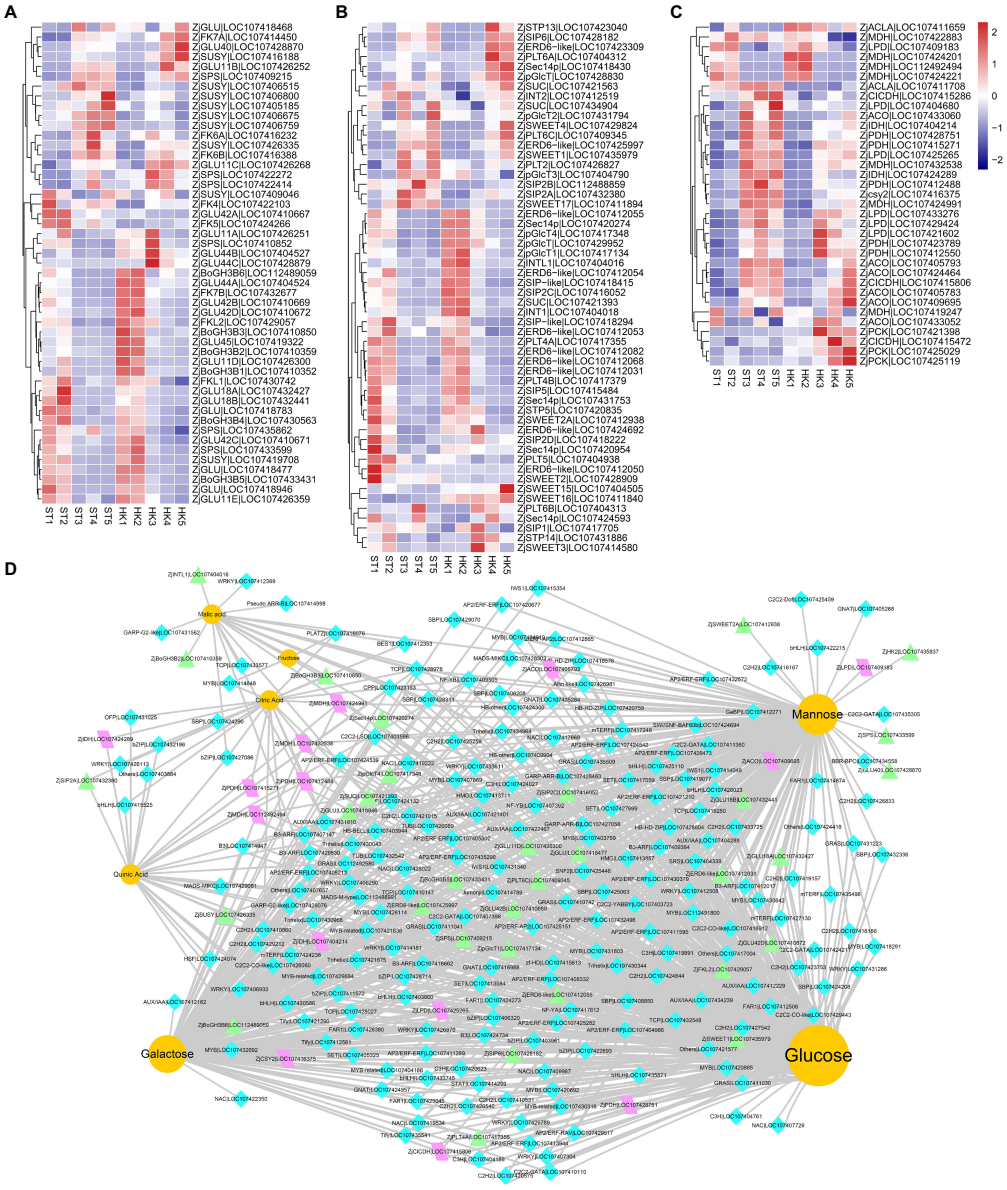
The expression profile of genes involved in organic acid and sugar biosynthetic pathways. Expression patterns of sugar-related kinases **(A)**, sugar transporters **(B)**, and tricarboxylic acid biosynthesis structural genes **(C)** during jujube ripening. The expression levels were standardized by *Z*-score. **(D)** The regulatory network of key flavor metabolites in jujube fruit. Yellow circles represent sugars and organic acids, turquoise diamonds represent transcription factors, purple parallelograms represent structural genes of the TCA cycle, and green triangles represent sugar biosynthesis-related genes.

The analysis of genes related to sugar biosynthesis and transport and organic acid metabolism is of great significance to analyze and understand the accumulation of sugars and organic acids. During the 2/3 stage of jujube fruit development, more DEGs and DAMs were involved in the pathway of starch and sucrose metabolism and carbon metabolism (Supplementary Figure S4). In this study, eight fructokinases (*ZjFK*), nine sucrose synthases (*ZjSUSY*), 26 glucosidase-like (*ZjGLU*, four alpha-, 22 beta-), two hexokinases (*ZjHK*), six sucrose-phosphatases (*ZjSPS*), and six alactinol–sucrose galactosyltransferases (*ZjSIP*) were identified. Moreover, 45 sugar transporter genes were found, including three sucrose transport proteins (*ZjSUC*), three sugar transporters (*ZjSTP*), 10 ERD6-like sugar transporters (*ZjERD6*-like), eight SWEET sugar transporters (*ZjSWEET*), seven polyol transporters (*ZjPLT*), three inositol transporters (*ZjINT*), five phosphatidylinositol transfer proteins, four plastidic glucose transporters (*ZjpGlcT*), and two UDP-glucose transporters (Figures 6A,B).

Citric and malic acids are the main organic acids of jujube fruits, and they are also vital intermediates in the tricarboxylic acid cycle (TAC) downstream of glycolysis. In our analysis, 35 DEGs of the TAC pathway were found, including six aconitate hydratases (*ZjACO*), two ATP-citrate synthase alpha chain proteins (*ZjACLA*), one citrate synthase (*ZjCSY*), six dihydrolipoyl dehydrogenases (*ZjLPD*), two isocitrate dehydrogenase [NAD] catalytics (*ZjIDH*), three isocitrate dehydrogenase [NADP] (*ZjCICDH*), seven malate dehydrogenase (*ZjMDH*), three phosphoenolpyruvate carboxykinase (*ZjPCK*), and five pyruvate dehydrogenase E1 component subunit alpha (*ZjPDH*) (Figure 6C).

Transcriptional regulation is an important cause of gene expression and regulation of metabolite content. We jointly analyzed and screened transcription factors and structural genes related to sugars and organic acids. We found 3,118 genes associated with mannose, glucose, galactose, fructose, quinic acid, citric acid, and malic acid, including 202 transcription factors in 57 gene families distributed in modules turquoise, brown and red (Supplementary Table S4) (|coefficient| ≥ 0.85, *p* < 1.38 × 10^−12^). Therefore, a co-expression network of sugar and acid metabolites with transcription factors and metabolic pathway structural genes was further constructed (Figure 6D). The results showed that candidate genes such as *ZjHAP3* (HEME ACTIVATOR PROTEIN (YEAST) HOMOLOG 3, LOC107409505), *ZjTCP14* (TEOSINTE BRANCHED, CYCLOIDEA AND PCF14, LOC107428978), and *ZjAGL61* (AGAMOUS-LIKE 61, LOC112488991) (At2g24840) may be involved in the accumulation of major sugars and acids in jujube fruits.

To further verify the correctness of the transcriptome data, we selected 10 genes for qRT-PCR validation. The results showed that the transcriptome expression trends were consistent with the qRT-PCR results. *ZjTCP14*, *ZjYABBY1*, and *ZjSKU5* were highly expressed at the early stages of fruit development, while the other genes were highly expressed at late stages (Supplementary Figure S5).

## Discussion

The combined analysis of metabolomics and transcriptomics are important technical tools for studying the flavor and nutrition of fruits such as watermelon, apple and jujube (Xu et al., 2020; Gong et al., 2021a), but there is still a lack of comprehensive understanding of the accumulation patterns of sugars, acids, and substances at different development stages of jujube. To reveal fruit flavor differences between the two cultivars that differed in flavor due to the different sugar and acid accumulation, this study constructed a global metabolome dataset of the two cultivars at five periods to provide a basis for studying the molecular accumulation of jujube metabolites. We identified 437 DAMs and 16,245 DEGs during fruit ripening. There were some special metabolites, such as LPC and LPE, that were highly accumulated between the two cultivars in the later stage of HK, with high levels of amino acids (leucine, arginine, and homoarginine) in ST (Figures 3D,E). This may have contributed to the different nutritional values of the two cultivars.

Soluble sugars, organic acids, and volatiles are important attributes that determine the color, flavor, and economic value of fruits (Gong et al., 2021a). Jujube is the largest economic tree species in China. Organic acids and soluble sugars change dramatically during the process of fleshy fruits from young to full maturity. Consistent with previous studies, the major sugar components in jujube fruits are fructose, glucose, and sucrose, and organic acids including citric acid and quinic acid (Zhang et al., 2021; Zhao et al., 2021). In this study, we found that both dominant sugars and organic acids were high in the early stages of development and decreased during later stages (Figure 4). Unlike other research that suggested that malic acid was dominant in the later stages of fruit development (Zhen et al., 2016), citric acid was the main content of both cultivars at all stages of fruit development, and the content of quinic acid was higher than that of malic acid. Citric acid and succinic acid are the main factors affecting acidity.

Elucidating the underlying molecular mechanisms of sugar and organic acid changes and their spatiotemporal interactions is a crucial step in understanding fruit development (Yu et al., 2021). The fruit flavor is controlled by the environmental signaling pathways, developmental signaling pathways, metabolic signaling pathways, and transcription factors play important roles in these processes (Hanson et al., 2008; Bastías et al., 2011). Overexpression of *SlAREB1* (ABA-response element binding factors) promoted levels of citric acid, malic acid, glutamic acid, glucose, and fructose in tomato (Bastías et al., 2011). While in apple (*Malus domestica* Borkh.), *MdAREB2* promoted sucrose and soluble sugar accumulation by activating *MdSUT2* (sugar transporter) (Ma et al., 2017). *AcERF182* regulated *AcBAM3.5*, a key structural gene involved in soluble sugar accumulation in kiwifruit (*Actinidia chinensis* Planch) (Wang et al., 2022). *MdbHLH3* directly activated *MdcyMDH* to promote malic acid accumulation in the apple. Additionally, overexpression of *MdbHLH3* increased photosynthetic capacity and carbohydrate content in apple leaves and also increased carbohydrate accumulation in fruits by regulating carbohydrate distribution from source to sink (Yu et al., 2021). Frank et al. (2018) reported that *BASIC LEUCINE ZIPPER63* (*bZIP63*) affects the circadian rhythm of *Arabidopsis* in response to sugar changes by regulating PSEUDO *RESPONSE REGULATOR7* (*PRR7*). In this study, we analyzed the genes that may be related to sugar and organic acid metabolites through WGCNA and Person’s relation, and identified transcription factors such as *ZjYABBY1*, *ZjMYB78*, *ZjHAP3*, *ZjTCP14*, and *ZjAGL61*, which can be co-expressed with metabolites and related structural genes at the same time (Figures 5B,C, 6D).

In the present study, *ZjYABBY1*, *ZjHAP3* and *ZjAGL61*were identified as candidate genes regulating the accumulation and metabolism of sugars and organic acids, suggesting that they may participate in fruit development through the metabolic pathways of sugars and organic acids. It is known that fruit formation and ripening is a very complex process, many aspects of fruit size, shape, and further developmental changes depending on organ identities are determined at an early stage (Karlova et al., 2014), Therefore, genes that regulate the dynamic changes of sugar and acid contents during fruit ripening may also be related to fruit morphology. For example, the *ZjYABBY1* gene, which is related to sugar and acid metabolism in this study, has a homologue, *AtYABBY*, that functions in *Arabidopsis* flower as *CRABS CLAW* (*CRC*), which is involved in organ polarity in carpel and nectary development (Bowman and Smyth, 1999; Huang et al., 2013). Another *AtAGL61* regulates central cell development in *Arabidopsis*. MADS-domain proteins *TOMATO AGAMOUS-LIKE1* (*TAGL1*) and *MADS1* were found to be involved in fruit ripening in tomato (Itkin et al., 2009; Dong et al., 2013; Karlova et al., 2014). *ZjHAP3* is a homologous gene of *AtHAP3* (At2g38880), which controls the initiation and development of plant seed embryonic (Su et al., 2021). In contrast, previous studies have shown that *OsHAP3E* participated in the determination of meristem identity in both vegetative and reproductive developments of rice (Zhang and Xue, 2013). It was shown that *AtTCP14* (At3g47620) can break seed dormancy (Zhang et al., 2019; Ferrero et al., 2021). *ZjMYB78* functions in response to abscisic acid and plant drought stress (Dalal et al., 2018).

## Conclusion

In general, this study identified the differences in gene expression and nutrient accumulation in different developmental stages of jujube through transcriptome and metabolome analysis. The accumulation of sugars and acids showed opposite trends. Several transcriptional regulators that may affect fruit flavor (sugar and acid) accumulation were identified by joint analysis. The mining of these candidate regulatory genes provides a basis for further improving the flavor and economic value of jujubes.

## Data availability statement

The data presented in the study are deposited in the SRA repository, accession number PRJNA835207.

## Author contributions

QP and YZ designed the research and revised the manuscript. DL and LZ conducted experiments and data analysis and wrote the manuscript. DL, LZ, YW, and PL performed data analysis. All authors contributed to the article and approved the submitted version.

## Funding

This study was supported by the National Key R&D Program of China (2019YFD1001605), Beijing Postdoctoral Research Foundation (2022-ZZ-107), the Special Fund for the Construction of Scientific and Technological Innovation Capability (KJCX20200114), the Key R&D Program of Hebei Province (20326807D), and the Key Science and Technology Program of Inner Mongolia Autonomous Region (2021ZD0041-004).

## Conflict of interest

The authors declare that the research was conducted in the absence of any commercial or financial relationships that could be construed as a potential conflict of interest.

## Publisher’s note

All claims expressed in this article are solely those of the authors and do not necessarily represent those of their affiliated organizations, or those of the publisher, the editors and the reviewers. Any product that may be evaluated in this article, or claim that may be made by its manufacturer, is not guaranteed or endorsed by the publisher.

## Footnotes

^1^ www.metware.cn
