# Supplementary figures and images for: An integrated metabolome and transcriptome approach reveals the fruit flavor and regulatory network during jujube fruit development
Source: Front Plant Sci. 2022 Sep 23;13:952698. doi: 10.3389/fpls.2022.952698 (PMC9537746; doi:10.3389/fpls.2022.952698)

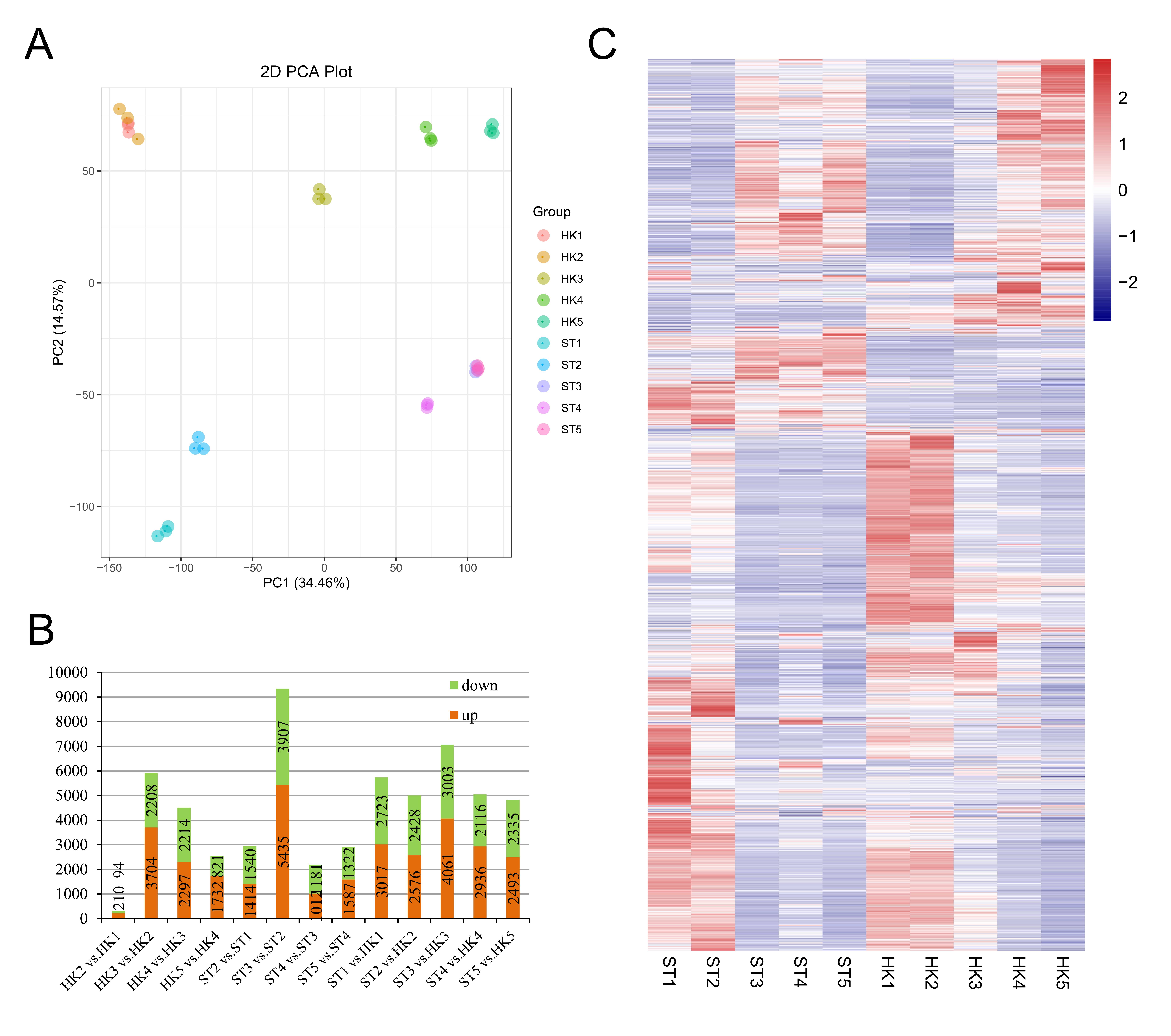

Supplement: Supplementary file 2 [file Image_1.JPEG]

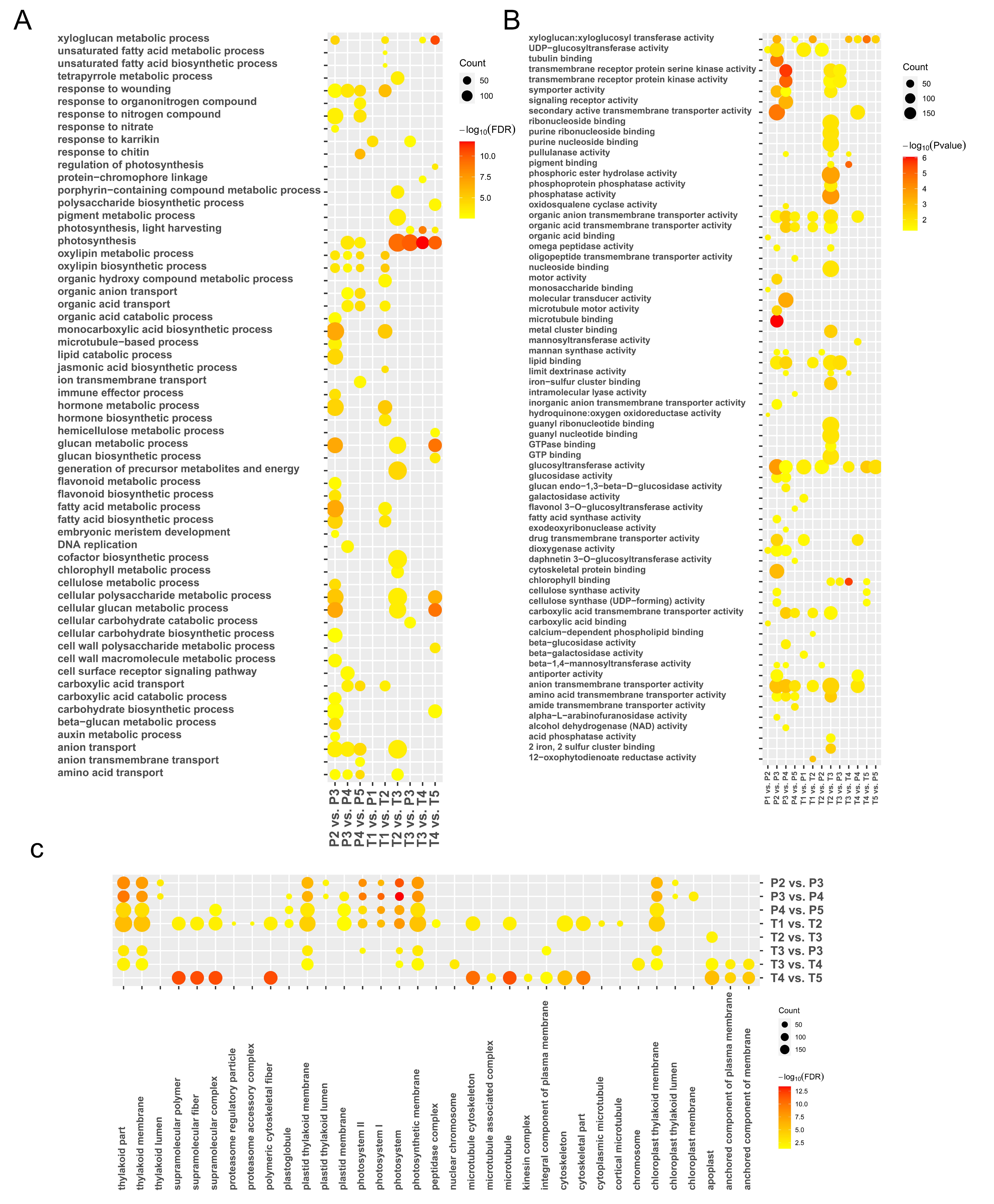

Supplement: Supplementary file 3 [file Image_2.JPEG]

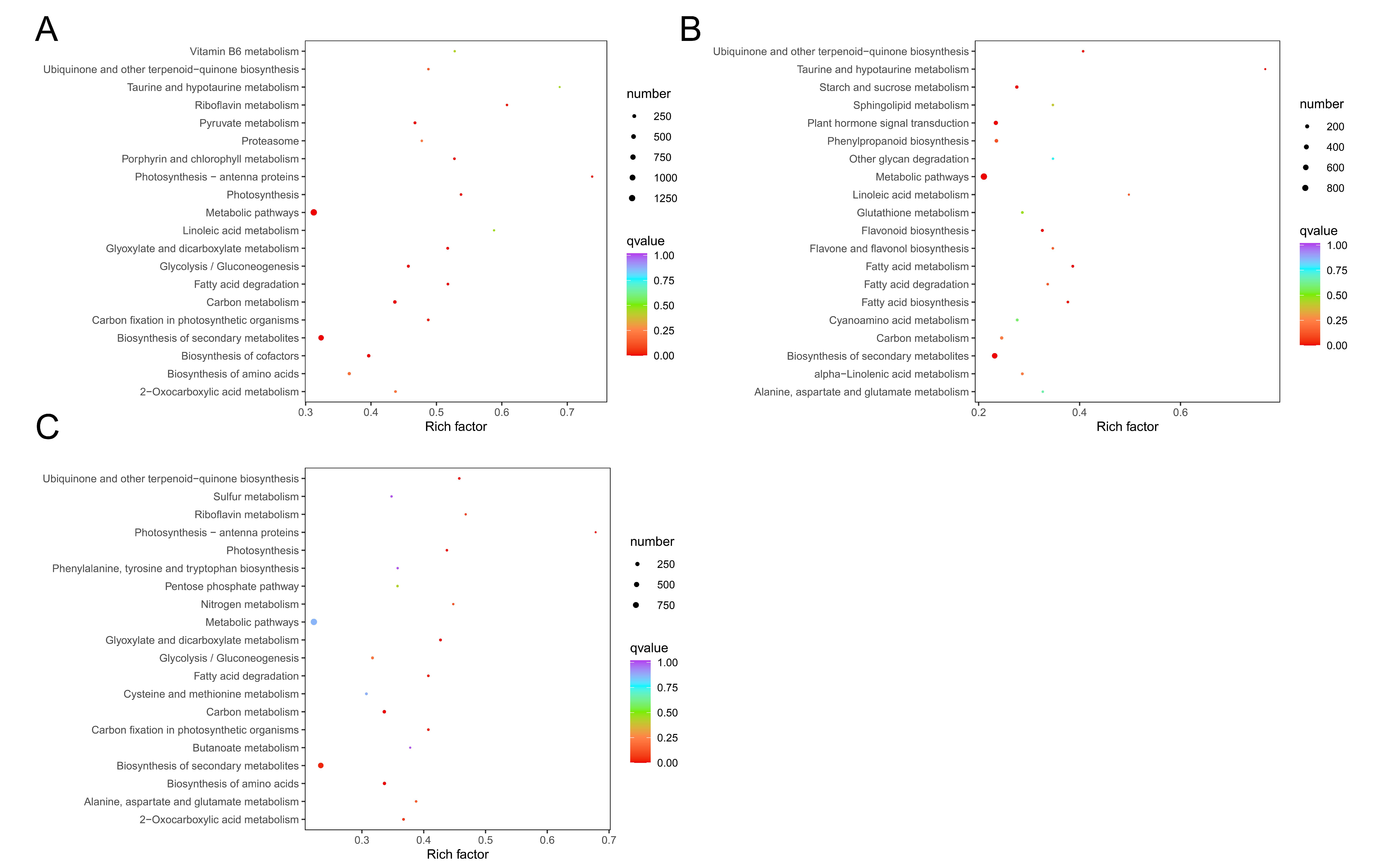

Supplement: Supplementary file 4 [file Image_3.JPEG]

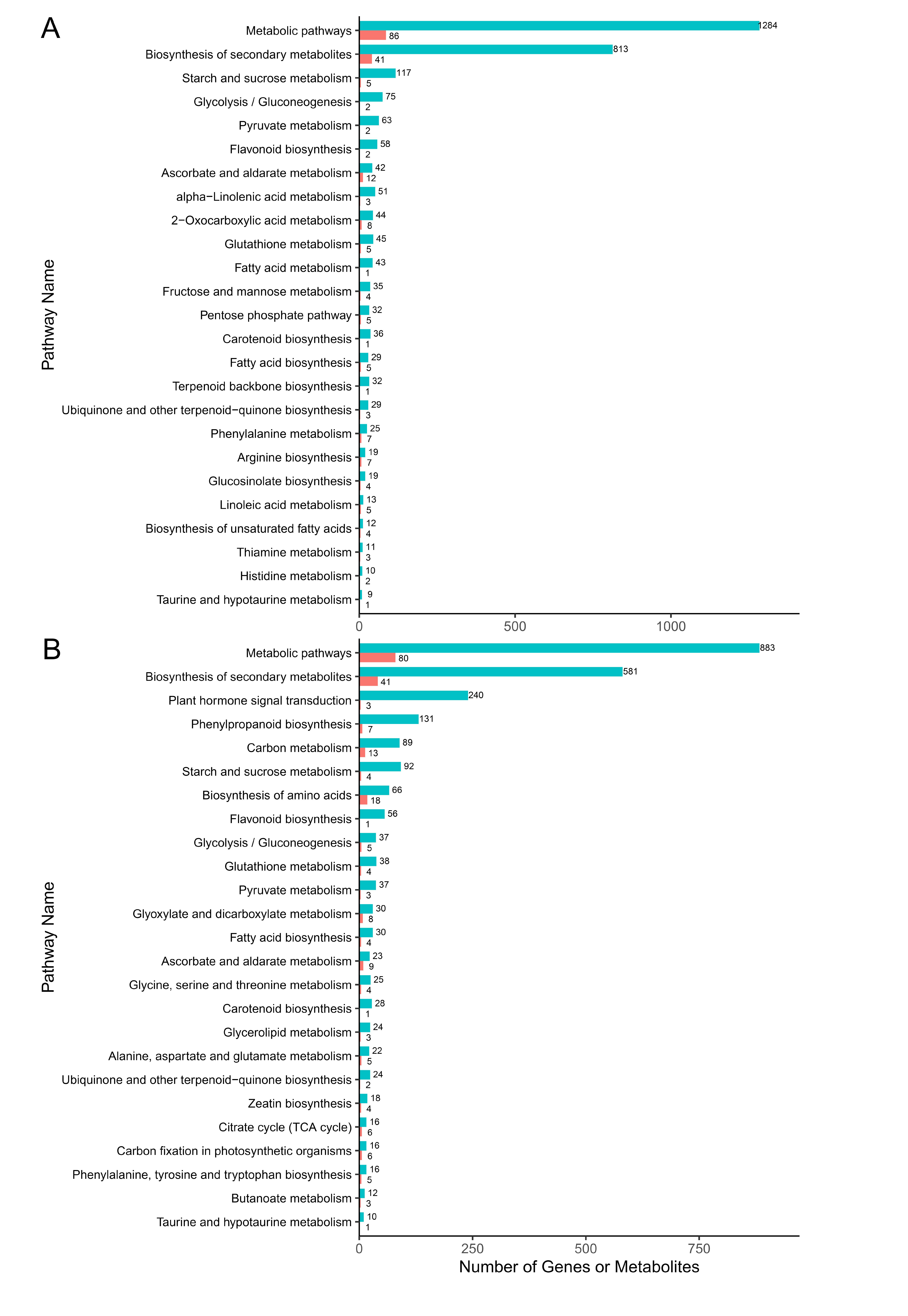

Supplement: Supplementary file 5 [file Image_4.JPEG]

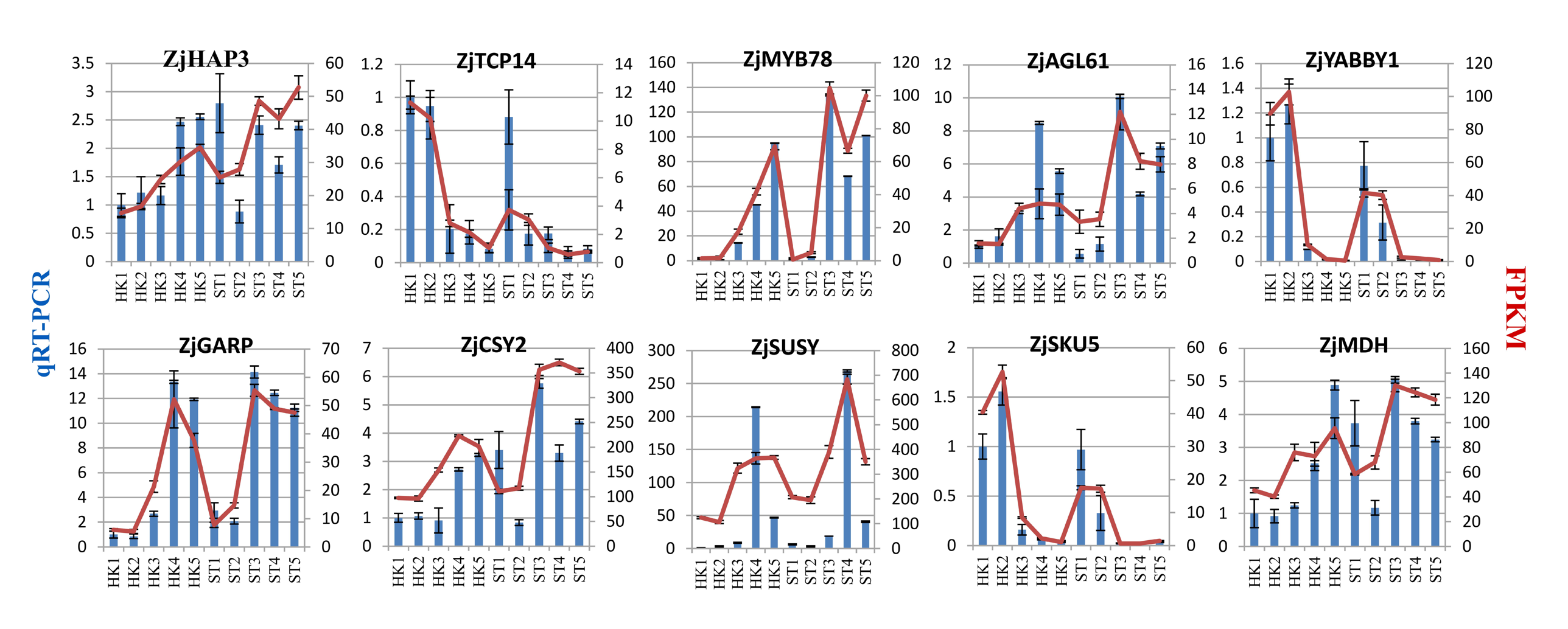

Supplement: Supplementary file 6 [file Image_5.JPEG]
